# Supplementary material for: Time of Day of Vaccination Affects SARS-CoV-2 Antibody Responses in an Observational Study of Health Care Workers
Source: J Biol Rhythms. 2021 Dec 4;37(1):124–9. doi: 10.1177/07487304211059315 (PMC8825702; doi:10.1177/07487304211059315)
Supplement: sj-docx-1-jbr-10.1177_07487304211059315 – Supplemental material for Time of Day of Vaccination Affects SARS-CoV-2 Antibody Responses in an Observational Study of Health Care Workers [file sj-docx-1-jbr-10.1177_07487304211059315.docx]

Supplemental Figure 1:


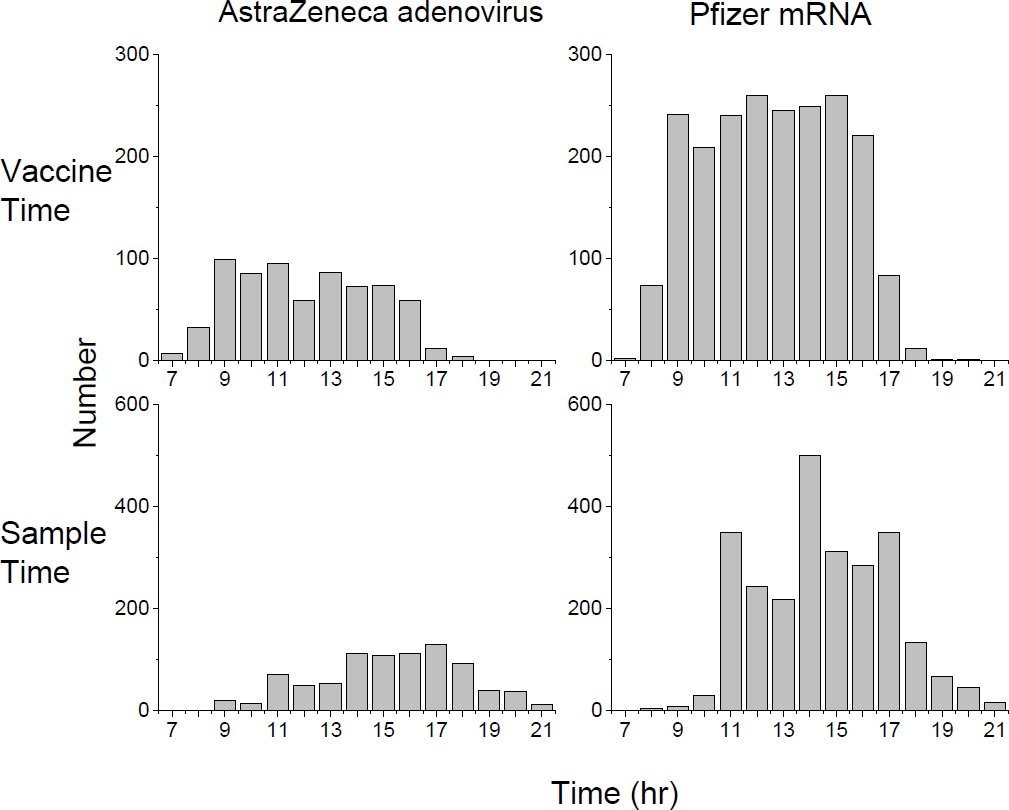


Histograms showing time (hour) of day of vaccinations (N=2784) and of sample collection (N=3425)
