# Supplementary material for: Time of Day of Vaccination Affects SARS-CoV-2 Antibody Responses in an Observational Study of Health Care Workers
Source: J Biol Rhythms. 2021 Dec 4;37(1):124–9. doi: 10.1177/07487304211059315 (PMC8825702; doi:10.1177/07487304211059315)
Supplement: sj-docx-2-jbr-10.1177_07487304211059315 – Supplemental material for Time of Day of Vaccination Affects SARS-CoV-2 Antibody Responses in an Observational Study of Health Care Workers [file sj-docx-2-jbr-10.1177_07487304211059315.docx]

Supplemental Figure 2

**Age: 16–29 Age: 30–39**

10,000


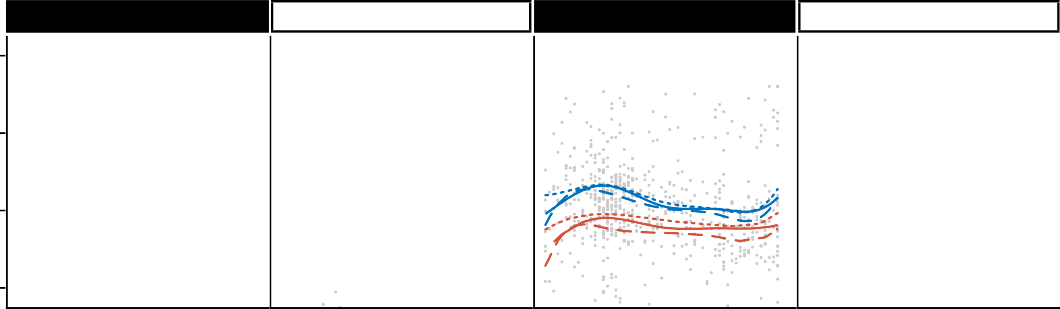

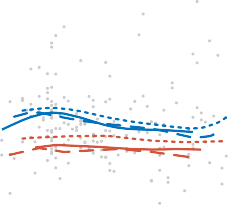

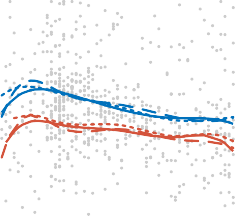

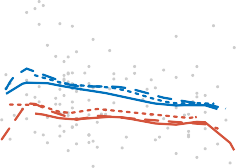


**Female**

**Male**

**Female**

**Male**

1,000

100

Abbott anti-spike reading

10

**Age: 40–49 Age: 50–74**

10,000


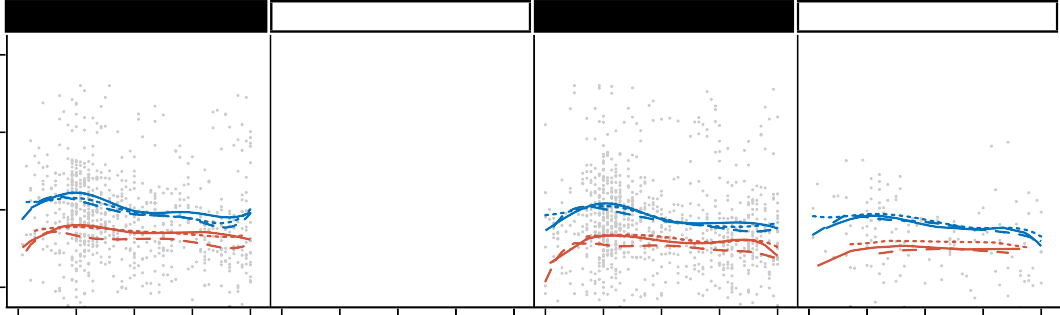

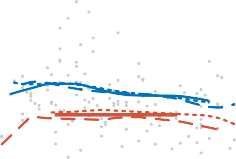


**Female**

**Male**

**Female**

**Male**

1,000

100

10

2 4 6 8 10

2 4 6 8 10

2 4 6 8 10

2 4 6 8 10

Weeks since 1st vaccine

Pfizer, Time 1 Pfizer, Time 2 Pfizer, Time 3

AstraZeneca, Time 1 AstraZeneca, Time 2 AstraZeneca, Time 3

B-spline fit curves, including time-of-day of vaccination (Time 1, 07:00-10:59; Time 2, 11:00- 14:59; Time 3, 15:00-21:59), vaccine type, age group, sex, and days post vaccination to log_10_ (Anti-Spike antibody response) Data estimates are split by time-of-day of vaccination vaccine type (Pfizer mRNA, blue; AstraZeneca Adenovirus, red), age group, and sex (Female or Male).
