# Supplementary material for: Time of Day of Vaccination Affects SARS-CoV-2 Antibody Responses in an Observational Study of Health Care Workers
Source: J Biol Rhythms. 2021 Dec 4;37(1):124–9. doi: 10.1177/07487304211059315 (PMC8825702; doi:10.1177/07487304211059315)
Supplement: sj-pdf-3-jbr-10.1177_07487304211059315 – Supplemental material for Time of Day of Vaccination Affects SARS-CoV-2 Antibody Responses in an Observational Study of Health Care Workers [file sj-pdf-3-jbr-10.1177_07487304211059315.pdf]

Supplemental Table 1: Type III tests of fixed effects from mixed effects model with sample time

Effect	Num DF	F	Probability
Main Effects			
Vaccination_Time<br>(Time 2, Time 3 vs Time) [‡]	2	4.40	0.0123
Sample_Time<br>(Time 2, Time 3 vs Time 1) [‡]	2	2.34	0.0969
Vaccine type<br>(AstraZeneca vs. Pfizer)	1	150.68	<0.0001
Age<br>(30-39, 40-49, 50-74 vs.16-29)	3	51.76	<0.0001
Sex<br>(Female vs. Male)	1	6.02	0.0142
Days post-vaccination	6	19.24	<0.0001
Interaction terms			
Days*Vaccination_Time	6	1.28	0.2230
Days*Vaccine type	6	7.26	<0.0001
Days*Age	18	1.73	0.0283
Days*sex	6	1.09	0.3684
Vaccination_Time*Vaccine type	1	1.23	0.2926
Vaccination_Time*Age	3	0.71	0.6387
Vaccination_Time*Sex	1	0.42	0.6604

Details of the linear mixed modeling are: Time of vaccination (Time 1, 07:00-10:59; Time 2, 11:00-14:59; Time 3, 15:00-20:59), vaccine type (Pfizer or AstraZeneca), age groups (from Table 1A), sex, and days post-vaccination were treated as fixed factors. A B-spline transformation of days post-vaccination was used to model the non-linear pattern of anti-Spike antibody responses (log10 transformed) post vaccination.
DF= Degrees of Freedom.† For all F tests the denominator DF was 3357. ‡ For each F test, the fixed effect referent is the last term shown, the F and P values are the Type III tests of overall fixed effects.
